# Conjunctival and corneal reactions in rabbits following short- and repeated exposure to preservative-free tafluprost, commercially available latanoprost and 0.02% benzalkonium chloride

**DOI:** 10.1136/bjo.2008.138768

**Published:** 2008-08-22

**Authors:** H Liang, C Baudouin, A Pauly, F Brignole-Baudouin

**Affiliations:** 1 Department of Toxicology, Faculty of Biological and Pharmacological Sciences, University Paris Descartes, Paris, France; 2 INSERM UMR S 592, Institute of Vision, University of Paris 6, Paris, France; 3 Department of Ophthalmology III, Quinze-Vingts National Ophthalmology Hospital, Paris, France; 4 Ambroise Paré Hospital, APHP, University of Versailles, Paris, France

## Abstract

**Aim:**

To compare the conjunctival and corneal reactions of commercially available solution of latanoprost (Xalatan) and preservative-free (PF) tafluprost in rabbits.

**Methods:**

The rabbits received 50 μl of phosphate-buffered saline (PBS), PF-tafluprost 0.0015%, latanoprost 0.005% or benzalkonium chloride (BAK) 0.02%; all solutions were applied at 5 min intervals for a total of 15 times. The ocular surface toxicity was investigated using slit-lamp biomicroscopy examination, flow cytometry (FCM) and on imprints for CD45 and tumour necrosis factor-receptor 1 (TNFR1) conjunctival impression cytology (CIC) and corneal in vivo confocal microscopy (IVCM). Standard immunohistology also assessed inflammatory/apoptotic cells.

**Results:**

Clinical observation and IVCM images showed the highest ocular surface toxicity with latanoprost and BAK, while PF-tafluprost and PBS eyes presented almost normal corneoconjunctival aspects. FCM showed a higher expression of CD45+ and TNFR1+ in latanoprost- or BAK-instilled groups, compared with PF-tafluprost and PBS groups. Latanoprost induced fewer positive cells for inflammatory marker expressions in CIC specimens compared with BAK-alone, both of which were higher than with PF-tafluprost or PBS. Immunohistology showed the same tendency of toxic ranking.

**Conclusion:**

The authors confirm that rabbit corneoconjunctival surfaces presented a better tolerance when treated with PF-tafluprost compared with commercially available latanoprost or BAK solution.

Prostaglandins (PGs) occupy centre stage among glaucoma medications.^1^ Currently, four different PG analogues—isopropyl unoprostone, latanoprost, travoprost and bimatoprost—are used for the treatment of glaucoma. These four PGs are superior to beta-adrenoceptor antagonists in terms of lowering intraocular pressure (IOP), and they have no severe side effects during long-term clinical use.^2^ ^3^ Benzalkonium chloride (BAK) is the most commonly used preservative in eye-drops. However, during in vitro and ex vivo studies, eye-drops containing different doses of BAK had an effect on the ocular surface, possibly due to the presence of BAK.^4^^–^6 In cell culture, it was seen that BAK induced epithelial cell death,^7^ pro-inflammatory or pro-apoptotic mediators,^8^ and oxidative stress, including mitochondrial activity and glutathione injury.^9^ On the ocular surface of patients with glaucoma, BAK induced complex inflammatory mechanisms, causing both allergy and toxicity.^4^

New PG formulations without BAK and without preservatives are currently in development. One such example is travoprost Z, in which BAK has been replaced with the Sofzia preservative system. This is an Alcon patent containing borate, sorbitol, propylene glycol and zinc chloride as a microbial-contamination-prevention system. Exempt of preservative, it might contribute to a better behaviour on the ocular surface.^10^ ^11^ In vitro studies using Chang conjunctiva-derived cells have shown that travoprost Z presents no pro-apoptotic or oxidative stress effects.^11^ Tafluprost is a newly synthesised PG F_2α_-agonist currently in development.^12^ ^13^ Tafluprost is highly selective for FP receptors and is a more potent agonist than latanoprost.^12^^–^14 In vitro studies have shown a reduction in toxicity with preservative-free (PF) tafluprost in human conjunctival epithelial cell lines.^15^

We developed in vivo tools for the analysis of experimental toxic models consisting of in vivo confocal microscopy (IVCM) and flow cytometry (FCM) evaluation of conjunctival impression cytology (CIC).^16^^–^20 The combination of these two in vivo and ex vivo tools is significant as they are reliable for the observation, at a cellular level, of toxic and/or immunoallergic reactions on the ocular surface, and can analyse inflammation and apoptosis in the conjunctival epithelium without the need for sacrificing the test animal. In this study, we have combined these two techniques to evaluate the toxicological profiles of PF-tafluprost, commercially available latanoprost and 0.02% BAK, which is the concentration of BAK used in the commercial solution of latanoprost.

## MATERIALS AND METHODS

### Animals and eye-drop treatments

Adult male New Zealand albino rabbits weighing 2.5–3.0 kg were treated according to the guidelines issued by the Association for Research in Vision and Ophthalmology. Before all experiments, rabbits were all anaesthetised.

In total, 24 rabbits were divided into four groups. Each group was composed of six rabbits: five rabbits were used for clinical and IVCM observations at 4 h (H4) and day (D) 1 to D7 after the administration of the treatment, and the remaining rabbit was sacrificed for immunohistological procedures at D1, a time point chosen for the maximal inflammatory infiltration time as a previous study^18^ and IVCM observation. Eye-drops were instilled according to the model described by Ichijima *et al*.^21^ Each of the four groups of rabbits was given a different treatment: 50 μl of phosphate-buffered saline (PBS); PF-tafluprost 0.0015% ophthalmic solution (Santen Oy, Tampere, Finland), commercially available latanoprost 0.005% ophthalmic solution (Xalatan, Pfizer, New York) or PBS containing 0.02% BAK. All solutions were applied using a micropipette to both eyes of the rabbits at 5 min intervals for a total of 15 times.

### Clinical findings and Draize test

The time was noted when obvious redness occurred on the ocular surface, calculated from the first instillation. Eyes were examined by slit-lamp biomicroscopy and were scored according to a modified Draize test.^16^^–^18

### IVCM evaluation

The Heidelberg Retina Tomograph II/Rostock Cornea Module (Heidelberg Engineering GmbH, Heidelberg, Germany) laser-scanning IVCM was used to examine the rabbits.^16^^–^18 22 IVCM scores were used to evaluate ocular surface toxicity profiles in four histological zones (table 1).^18^

**Table 1:** In vivo confocal microscopy scoring for the evaluation of ocular toxicity in the cornea, limbus and conjunctiva (maximum score: 40)

Eye zone	Toxicity seen	In vivo confocal microscopy score
Superficial epithelium (max points 10)	Desquamation:	2
	Partial	
	Total important	4
	Shape/size: anisocytosis, microcytosis, macrocytosis, irregular shape, oedematous cells, swollen cells, loss of cell borders	2
	Reflectivity: abnormal reflectivity patterns, hyper-reflective cells, nuclei not visible in hyper-reflective cells	2
	Inflammation: presence of inflammatory infiltration	2
Basal epithelium (max 10 points)	Disorganisation	2
	Inflammatory infiltration:	2
	0>slight>100 cells/mm^2^	
	50>moderate>100 cells/mm^2^	4
	100>moderate>200 cells/mm^2^	6
	Severe>200 cells/mm^2^	8
Anterior stroma (max 10 points)	Disorganisation	2
	Inflammatory infiltration:	
	0>slight>100 cells/mm^2^	2
	50>moderate>100 cells/mm^2^	4
	100>moderate>200 cells/mm^2^	6
	Severe>200 cells/mm^2^	8
Limbus and conjunctiva (max 10 points)	Presence of capillary bud from limbal vessels (tendency toward neovascularisation)	2
	Presence of inflammatory infiltrates, rolling in limbal vessal/conjunctiva zone	2
	0>slight>50 cells/mm^2^	
	50>moderate>100 cells/mm^2^	4
	100>mild>200 cells/mm^2^	6
	Severe>200 cells/mm^2^	8

### Cresyl violet staining and FCM analysis in CIC

CIC specimens were collected as in previous studies for further cresyl violet cytology (1%, no. 5235, Merck, Fontenay sous bois, France) or FCM (FC500, Beckman Coulter, Miami, FL) procedures.^16^ ^18^^–^20 Membranes of 0.2 µm porosity were applied to the superior bulbar conjunctiva: two in nitrocellulose for cytology (Millipore, Bedford, MA) and two in polyether sulfone (Supor −200 Pall Life Sciences, Ann Arbor, MI) for FCM procedures. We evaluated the CIC according to a modified Nelson classification.^23^ Direct and indirect immunofluorescence procedures were used by FCM to study the expressions of tumour necrosis factor-receptor 1 (TNFR1; 1:40 dilution, R&D Systems, Minneapolis, MN) and rabbit CD45 (1:50, CBL1412, Cymbus Biotechnology, Chandlers Ford, UK). CD45 (leucocyte common marker) and TNFR1 have been used to assess inflammatory and/or toxicological phenomenon in animal models.^16^ ^18^

### Cryosections and immunohistology

Cryosections of enucleated eyes at D1 were prepared and incubated with antibodies to CD45/TUNEL assay (Roche Diagnostics, Meylan, France) to detect inflammatory/apoptotic cell infiltration, and counted using a fluorescent microscope (Olympus BX-UCB, Olympus, Melville, NY) equipped with a DP70 Olympus digital camera.

### Statistical analysis

Results were expressed as mean (SE). For Draize and IVCM scores, comparisons among the groups were performed by non-parametric comparisons (Mann–Whitney U test). Comparisons among the groups for CIC expressions and immunopositive cells counts were performed by factorial analysis of variance (ANOVA) followed by the Fisher method for pairwise comparisons (Statview V; SAS Institute, Cary, NC).

## RESULTS

### Clinical findings

**Figure 1:**
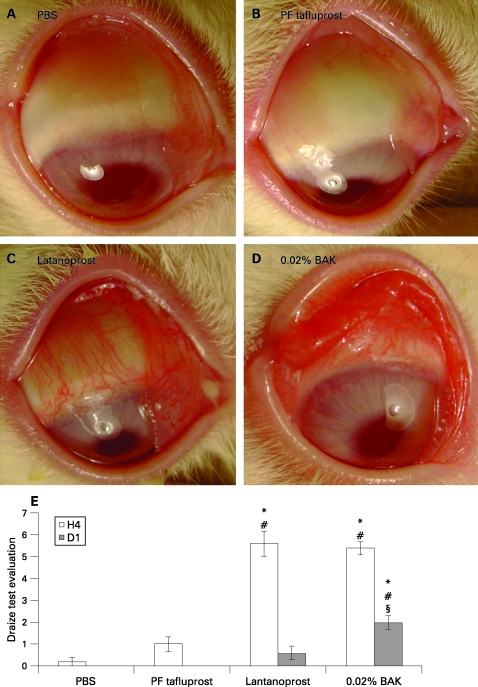
Photographs of rabbit eyes 4 h after the 15 instillations of phosphate-buffered saline (A: PBS), preservative-free tafluprost (B: PF tafluprost), latanoprost (C) or 0.02% benzalkonium chloride (D: BAK). (E) Draize test scores at H4 and D1 after treatment instillation. *p*<*0.005 compared with PBS; #p*<*0.01 compared with PF-tafluprost; §p*<*0.05 compared with latanoprost.

PBS and PF-tafluprost groups (fig 1A,B) presented the same aspects as found in normal rabbit eyes without any obvious abnormality. However, the latanoprost (fig 1C) and BAK (fig 1D) groups induced diffuse hyperaemia, chemosis and secretions on the conjunctiva. Latanoprost and BAK induced conjunctival redness very quickly after the first instillation (16.3 (SE 0.82) min and 13.5 (1.07) min, respectively). Tafluprost presented a slight and late redness (48.2 (3.83) min; p*<*0.0001 compared with BAK and latanoprost groups).

The PF-tafluprost group showed no statistical difference in ocular Draize scores compared with the PBS group (fig 1E). At H4, latanoprost and BAK showed higher Draize scores than PBS (p*<*0.005 for both) and PF-tafluprost (p*<*0.01 for both) groups. At D1, the latanoprost group returned to a normal aspect without any statistical differences compared with the PBS or PF-tafluprost groups. However, ocular changes were still observed at D1 for BAK with higher scores than for PBS (p*<*0.005), PF-tafluprost (p*<*0.01) and even latanoprost (p*<*0.05).

### IVCM images and IVCM score

#### Superficial epithelium

At D1 after instillation, PBS- and PF-tafluprost-instilled rabbits presented almost normal aspects of the corneal epithelium, with a regular polygonal mosaic appearance, brightly reflective nuclei and no obvious desquamation or swelling (fig 2.1A,B). Latanoprost and BAK solutions induced various pathological aspects of the corneal epithelium including partial desquamation of epithelial cells, irregular cell shapes, anisocytosis and loss of cell borders, abnormal reflectivity patterns, swollen cells and inflammatory infiltration (fig 2.1C,D).

**Figure 2:**
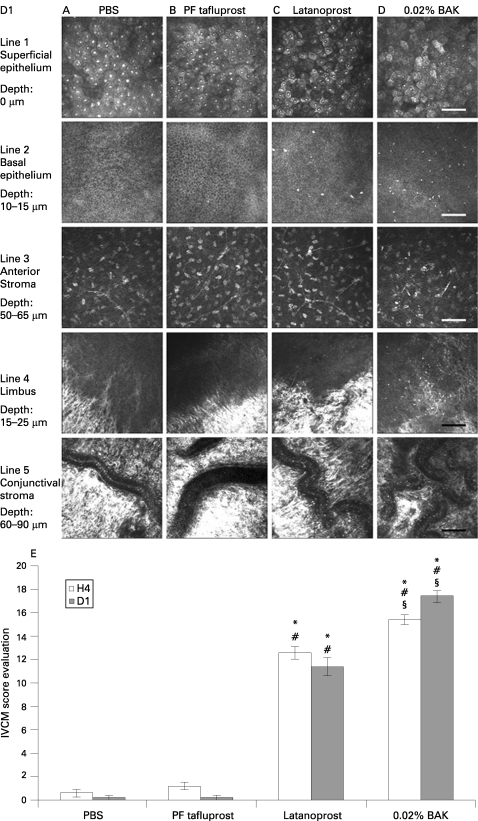
In vivo confocal images of rabbit corneal superficial epithelium (line 1), basal epithelium (line 2), anterior stroma (line 3), limbus (line 4) and conjunctival stroma (line 5) after instillations of: phosphate-buffered saline (A: PBS), preservative-free tafluprost (B: PF-tafluprost), latanoprost (C) or benzalkonium chloride (D: BAK), at D1. The scale bar indicates 100 μm. (E) In vivo confocal microscopy (IVCM) score evaluation at H4 and D1 after treatment instillation. *p*<*0.0005 compared with PBS; #p*<*0.0005 compared with PF-tafluprost; §p*<*0.005 compared with latanoprost.

#### Basal epithelium

PBS (fig 2.2A) and PF-tafluprost (fig 2.2B) did not induce any inflammation. Latanoprost (fig 2.2C) and BAK (fig 2.2D) induced important inflammatory cell infiltration but at significantly different levels (H4: 52 (5.32) cells/mm^2^ for latanoprost vs 106 (9.17) cells/mm^2^ for BAK, p*<*0.0001; and D1: 53 (7.95) cells/mm^2^ for latanoprost vs 130 (13.38) cells/mm^2^ for BAK, p*<*0.0001).

#### Anterior stroma

We observed a slight inflammatory infiltration and disorganisation only after instillations of BAK (fig 2.3D).

No abnormalities of the posterior stroma or endothelium were observed with any treatment (data not shown).

#### Limbus

No obvious responses were seen in the PBS and PF-tafluprost groups (fig 2.4A,B). In the latanoprost (fig 2.4C) and BAK (fig 2.4D) groups, we observed obvious inflammatory infiltrations in the peripheral cornea and the limbus area.

#### Conjunctival stroma

Both the PBS and PF-tafluprost instilled rabbits presented normal conjunctival blood vessels without any rolling inflammatory cells (fig 2.5A,B). In the latanoprost (fig 2.5C) and BAK (fig 2.5D) groups, obvious rolling of inflammatory cells was recorded.

The IVCM score evaluation to quantify the whole ocular surface abnormalities showed no significant differences at H4 and D1 between PF-tafluprost and PBS (fig 2E). Latanoprost and BAK showed higher IVCM scores compared with both PBS and PF-tafluprost (p*<*0.0005 for all comparisons). Interestingly, latanoprost induced lower IVCM scores than did BAK alone (p*<*0.005) at both H4 and D1. At D4, IVCM scores remained the highest after BAK-alone application (p*<*0.01 compared with the three other groups; data not shown). However, there were no differences in IVCM scores between latanoprost, PBS or PF-tafluprost groups. At D7, BAK-alone treated eyes returned to normal IVCM scores (data not shown).

### Cresyl violet staining and FCM analysis on CIC

**Figure 3:**
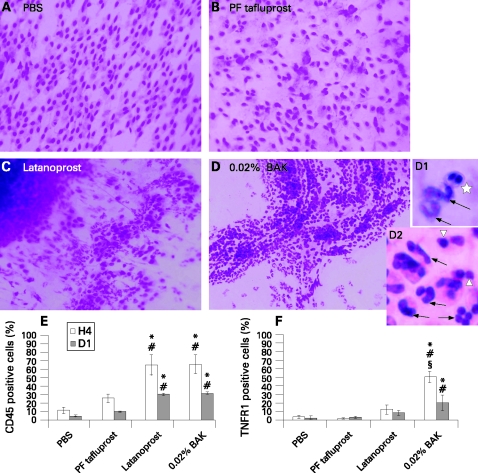
Cresyl violet-stained conjunctival impression cytology from rabbits instilled with phosphate-buffered saline (A: PBS), preservative-free tafluprost (B: PF-tafluprost), latanoprost (C) or 0.02% benzalkonium chloride (D: 0.02% BAK) (original size × 40). Conjunctival impression cytology expressions of (E) CD45+ and (F) tumour necrosis factor-receptor 1 (TNFR1) evaluated by flow cytometry. *p*<*0.05 compared with PBS; #p*<*0.05 compared with PF-tafluprost; §p*<*0.05 compared with latanoprost.

CIC specimens from rabbit eyes instilled with PBS showed a homogeneous cell sheet (fig 3A, table 2): the epithelial cells were flat and regular, with a nucleo:cytoplasmic ratio from 1:2 to 1:3. Goblet cells were clearly visible among or beside the epithelial cells. PF-tafluprost (fig 3B) instillation induced slight anisocytosis in the epithelium with normal nuclei. Slight infiltration of inflammatory cells could also be observed. The goblet cell number and morphology remained normal. Significant inflammatory infiltration was observed after the instillation of latanoprost (fig 3C) and BAK (fig 3D). In latanoprost-receiving eyes, a few conjunctival epithelial cells could still be observed, adjacent to inflammatory patches, with important anisocytosis and anisonucleocytosis. In BAK-receiving eyes, patches of inflammatory cell-containing secretions without any epithelial cells were observed. The abundant inflammatory cell infiltrates consisted mainly of neutrophils (polymorphonuclear cells) (arrows), lymphocytes (arrowheads) and rare eosinophils (star) (fig 3D1,D2). No goblet cells could be seen in these conditions.

**Table 2:** Description of cresyl violet impression cytology at 4 h

	Phosphate-buffered saline	Preservative-free tafluprost	Latanoprost	0.02% benzalkonium chloride
Nucleo:cytoplasmic ratio	1:2–1:3	1:2–1:3	Cell borders not clear	No epithelial cells visible
			Ratio non-applicable	
Anisocytosis/anisonucleocytosis	–	+	+++	No epithelial cells visible
Cell size	Normal	Normal	Very irregular	Non-applicable
Inflammatory cells	–	++	++++	++++
Goblet cells	Normal	Normal	Goblet cell imprint, no more complete cell	Could not been seen
Goblet cells:epithelial cells ratio, %	14	15	0	0

–, no abnormality; +, discrete; ++, moderate; +++, important; ++++, extremely intense.

At H4, PBS and PF-tafluprost induced 11.38 (3.68)% and 25.36 (4.98)% of CD45+ cells (fig 3E), respectively, with no difference. Latanoprost and BAK groups induced 65.38 (12.58)% and 65.95 (11.06)%, respectively, both of which were significantly higher than PBS and PF-tafluprost instillations (p*<*0.05 for all comparisons). At D1, latanoprost- and BAK-instilled groups still showed a higher expression (30.58 (1.49)% and 31.73 (1.27)%) than did PBS or PF-tafluprost (p*<*0.05 for the two groups). At H4, TNFR1+ cells were also observed after the instillation of BAK (fig 3F) (50.35 (6.96)%; p*<*0.05 compared with all other groups) and latanoprost (12.43 (5.41)%; p*<*0.05 compared with BAK alone). There were no significant differences between PBS or PF-tafluprost instilled groups. At D1 after instillation, the strong expression of TNFR1 in the BAK-alone group decreased to 20.25 (8.87)%, but it was still significantly higher than in PBS or PF-tafluprost (p*<*0.05) instilled eyes.

### Immunohistology of CD45 and TUNEL positive cells in rabbit cryosections

**Figure 4:**
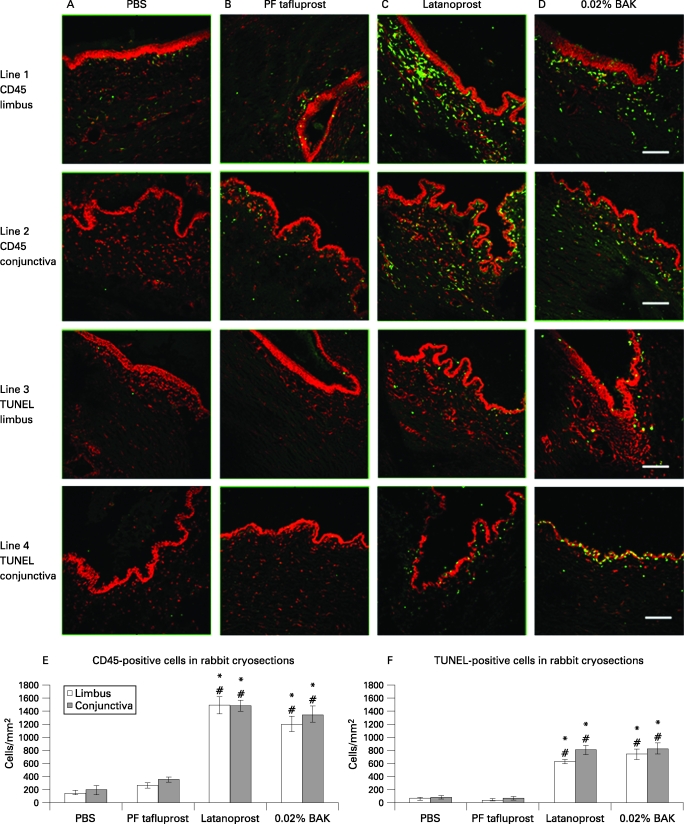
Immunohistology of CD45+ and TUNEL+ cells in rabbit cryosections at D1 after instillations of: phosphate-buffered saline (A: PBS), preservative-free tafluprost (B: PF-tafluprost), latanoprost (C) or benzalkonium chloride (D: BAK). The scale bars indicate 100 μm. (E) CD45+ and (F) TUNEL+ cell counts in limbus and conjunctiva of rabbit cryosections at D1 after eye-drop instillations. *p*<*0.0001 compared with PBS; #p*<*0.0001 compared with PF-tafluprost.

The immunostaining results of CD45+ and TUNEL+ cells at D1 are shown in fig 4A–D. The counts of positive cells were presented as cells/mm^2^ (fig 4E,F).

PBS and PF-tafluprost-instilled rabbit eyes showed only a few CD45+ inflammatory cells in the limbus (fig 4.1A,B) and conjunctiva (fig 4.2A,B) without any statistically significant differences. Latanoprost and BAK induced numerous CD45+ inflammatory cells in limbal and conjunctival areas: in the limbus, 1490 (132.04) cells/mm^2^ were found for latanoprost (fig 4.1C), and 1200 (115.47) cells/mm^2^ for BAK (fig 4.1D), with no differences. In the conjunctiva, 1480 (90.43) cells/mm^2^ were counted for latanoprost (fig 4.2C) and 1350 (121.34) cells/mm^2^ for BAK (fig 4.2D). The numbers of CD45+ cells were similar between latanoprost and BAK, and were significantly higher in both groups compared with the PBS (p*<*0.0001) and PF-tafluprost (p*<*0.0001) groups.

A similar trend was found for TUNEL+ apoptotic cells. Few TUNEL+ apoptotic cells were observed after instillation of PBS or PF-tafluprost instillations. For latanoprost, 630 (36.67)/mm^2^ TUNEL+ apoptotic cells in the limbus (fig 4.3C) and 810 (67.41)/mm^2^ in the conjunctiva (fig 4.4C) were seen (p*<*0.0001 compared with both PBS and tafluprost groups). Similar counts were found for BAK instillation (p*<*0.0001 compared with both PBS and tafluprost groups). These apoptotic cells were located especially in the epithelial layers and in the anterior stroma. In the conjunctiva, they were observed in particular abundance in the epithelial layers and also in the stroma.

## DISCUSSION

Fifteen repeated instillations of commercialised eye-drops can be considered a pertinent and sensitive model as it can distinguish between different levels of toxicity. Using in vivo tandem scanning confocal microscopy, this model was able to help distinguish between the toxicity ranking of 0.02%, 0.01% and 0.005% BAK concentrations by clearly showing the dose-dependent levels of epithelial deterioration.^21^ Without modifying the concentration and/or composition of the tested drugs, this present model of acute instillations appears useful for future drug toxicity evaluations because it combines rapidity and efficiency when comparing the toxicity of several products. Nevertheless, although convenient and rapid for between-drug comparisons, it should only be considered as an experimental model, as it is relatively aggressive and does not reflect real administration in patients. The clinical relevance of this experiment is that the lack of toxicity in this acute instillation model is a good indicator of further absence of ocular toxicity in a more conventional use in the long-term for glaucoma patients.

PF-tafluprost tested in this in vivo study presented almost similar results to PBS after clinical and IVCM observations, CIC analysis and inflammatory or apoptotic immunopositive cell counts in cryosections. Latanoprost solution and BAK alone were much more toxic than the PF-tafluprost solution. However, latanoprost instillations induced less toxicity than did BAK in rabbit ocular surface, especially with the Draize test evaluation at D1, IVCM scores at H4 and D1, and CIC analysed by FCM for TNFR1 at H4. PF-tafluprost was better tolerated on the rabbit ocular surface than the classic preservative-containing PG-analogue latanoprost solution. The good tolerability of PF-tafluprost is probably linked to the absence of preservative in the solution, and so we would speculate that a PF-latanoprost solution would have a similar tolerability on the ocular surface. Indeed, the main toxic effects of antiglaucoma treatments reported in the literature are related to the doses of preservative commonly used in eye-drop formulations.^4^^–^6 Topical antiglaucoma treatments with high concentrations of BAK are known to induce significant ocular surface changes, including tear-film drying,^24^ ^25^ thickening of corneal epithelium, the loss of goblet cells,^7^ inflammatory cell infiltration and proliferation in the conjunctiva,^7^ ^26^^–^28 and pro-apoptotic effects in conjunctival^9^ or trabecular meshwork cells.^29^

In eyes receiving PG analogues over the long-term, previous studies have shown low levels of inflammatory reactions compared with those seen with beta-adrenoceptor antagonists or multiple treatments, despite rather high concentrations of BAK in the commercial solutions.^4^ ^30^ Conversely, in toxicological studies carried out in conjunctival cell lines, the PG molecules seem to present a protective effect against BAK-induced toxicity.^5^ ^6^ ^30^ In this present in vivo model using 15 successive instillations in rabbit eyes, latanoprost also presented less toxicity than 0.02% BAK alone, especially in clinical criteria and CIC inflammatory marker expression.

The new PG analogue with a PF formulation, tafluprost, has shown promising results in clinical and experimental studies. In healthy males, the 0.0025% and 0.005% concentrations of tafluprost were generally well tolerated; tafluprost at 0.005% reduced IOP more than placebo or 0.005% latanoprost.^31^ In an in vitro study concerning immortalised epithelial cell line from normal human conjunctiva, treatment with PF-tafluprost resulted in a significantly higher membrane integrity, with lower pro-apoptotic and pro-oxidative effects when compared with commercially available solutions of latanoprost, travoprost and bimatoprost.^15^

The present toxicological study was based on a combination of original, technical, ex vivo and in vivo tools to explore the whole ocular surface. IVCM is well adapted to the transparent corneal tissues. Conversely, CIC are more suitable for conjunctival epithelium than corneal epithelium, as corneal epithelial cells are too cohesive and are difficult to detach and collect onto the membrane. Conjunctiva plays a role in ocular defence, which is an important tissue with which to explore the inflammatory aspects of deleterious effects induced by xenobiotics on the ocular surface.

We recommend this acute instillation model to assess the toxicity profiles of newly developed ophthalmic drugs. Our results showed that 0.02% BAK, chosen as a positive control of toxicity, was the most toxic agent tested. This was followed by latanoprost, which contains the same concentration of BAK but showed a tendency to be less toxic and confirms previous reports.^4^^–^6 Furthermore, we confirmed that the new antiglaucoma PG-analogue, PF-tafluprost, was very well tolerated as was PBS. Overall, the rabbit corneoconjunctival surface showed a better tolerance when treated with PF-tafluprost than with latanoprost or 0.02% BAK solution.
